# PolyJet-Printed Bellows Actuators: Design, Structural Optimization, and Experimental Investigation

**DOI:** 10.3389/frobt.2019.00034

**Published:** 2019-05-14

**Authors:** Gabriel Dämmer, Sven Gablenz, Alexander Hildebrandt, Zoltan Major

**Affiliations:** ^1^Institute of Polymer Product Engineering, Johannes Kepler University Linz, Linz, Austria; ^2^Advanced Mechatronic Systems, Festo AG & Co. KG, Esslingen am Neckar, Germany

**Keywords:** additive manufacturing, pneumatic actuator, printed robotics, pneumatic robot, multi-material 3D printing, visco-elastic, design for additive manufacturing (DfAM), structural optimization

## Abstract

Pneumatic bellows actuators are exceptionally suitable for Additive Manufacturing (AM) as the required geometrical complexity can easily be obtained and their functionality is not affected by rough surfaces and small dimensional accuracy. This paper is an extended version of a previously published contribution to the RoboSoft2018 conference in Livorno, Italy. The original paper (Dämmer et al., 2018) contains a simulation-driven design approach as well as experimental investigations of the structural and fatigue behavior of pneumatic multi-material PolyJet™ bellows actuators. This extended version is enhanced with investigations on the relaxation behavior of PolyJet bellows actuators. The presented results are useful for researchers and engineers considering the application of PolyJet bellows actuators for pneumatic robots.

## Introduction

The design of future robotic systems will be shaped by the demand for a large product variety and short lead times but may at the same time benefit from the constant progress in manufacturing technologies. In this context, multiple opportunities arise from the combination of AM and pneumatic actuation.

A possible approach to increase flexibility in future production processes are human-robot collaboration scenarios, characterized by the immediate proximity of humans and robots. The elimination of safety cages is accompanied by the obligatory demand of physical integrity of human co-workers. By minimizing manipulator link inertia and adding compliant elements in the kinematic chain, hazards inherent to robotic systems can be reduced (Zinn et al., 2004; DIN ISO/TS 15066:2017-04, 2017; Dämmer et al., 2018). AM technologies allow manufacturing of very complex geometries (Clausen et al., 2016) as those obtained by topology optimization, resulting in very light-weight parts. In electro-mechanical drive systems, inherent compliance comes at the cost of a significant increase in mechanical complexity (Lens et al., 2010). This applies in particular to inherently adjustable compliant systems (Grebenstein et al., 2011; Dämmer et al., 2018). Due to the compressibility of air, pneumatic actuators are inherently compliant and can easily be arranged to antagonistic pairs with adjustable compliance (Vanderborght et al., 2013; Baiden and Ivlev, 2014; Veale and Xie, 2016). Recently, the strive for double-acting pneumatic actuators has lead to novel designs that might result in very compact future products (Ferraresi et al., 2014). Moreover, functional integration enabled by AM (Paz et al., 2016) and mechanical simplicity of pneumatic actuators (Hildebrandt, 2009) may result in a reduced number of parts, therefore minimizing efforts for assembly and logistics. Additionally, in tool-less manufacturing technologies, such as AM, quantities have a reduced effect on manufacturing cost and therefore enhance flexible processes and low batch sizes. However, for the investigation of the expected benefits, suitability of future robotic components for AM is mandatory.

From the perspective of additive manufacturability, bellows actuators are very promising for two reasons. First: Good surface quality and dimensional accuracy that can hardly be achieved by AM technologies (MacCurdy et al., 2016) are not required for the bellows working principle. Second: The complex folded structure exploits the geometrical freedom inherent to AM and actuators performance can easily be modified by shape and material variations. An excellent example of functional integration by additive manufacturing is the Bionic Handling Assistant (Grzesiak et al., 2011) in which additively manufactured bellows structures accommodate external loads and move the robot.

For AM of bellows structures, various technologies have been used, such as selective laser sintering (SLS) (Grzesiak et al., 2011), PolyJet™ printing (PJP) (MacCurdy et al., 2016; Drotman et al., 2017) and Digital Mask Projection Stereolithography (DMP-SL) (Peele et al., 2015). AM of molds for silicone molding of bending actuators was demonstrated in numerous publications (e.g., Mosadegh et al., 2014). Also, detailed reviews of bellows actuators in the context of articulated robotic systems (Gaiser et al., 2012), 3D printing (Zolfagharian et al., 2016) and soft robotics (Polygerinos et al., 2017) have been published.

In the EU founded research project “Digital Materials for 3D Printing” (DIMAP), novel functional materials for PolyJet™ printing were developed (http://www.dimap-project.eu). As an application showcase, PolyJet-printable highly integrated robot components—including bellows actuators—were designed. However, despite of several examples of AM bellows actuators in the context of robotics, there is a lack of knowledge on the achievable performance and simulation-driven design strategies. In addition to that, repeated loadings have recently been related to a significant decrease of the sustainable strains in PolyJet elastomers (Moore et al., 2015).

In the conference paper (Dämmer et al., 2018), pneumatic multi-material PolyJet bellows actuators are presented. Their structural behavior is investigated and compared to structural simulations. In order to improve the actuators performance, bellows design is interpreted as a shape optimization problem with strain minimization as the main objective. Finally, endurance runs are performed to investigate the effects of shape optimization and two different materials on the number of load cycles to failure. Because force responses to imposed displacements were observed to be significantly time dependent, this extended version is enhanced with relaxation tests of elastomeric bellows structures. The relevance of the presented results for the development of a multi-material light-weight gripper is presented demonstrated in another extended version paper (Dämmer et al., 2019). The presented results contribute to the general knowledge concerning the use of PolyJet elastomers for pneumatic actuators and robots.

## Design

**Figure 1:**
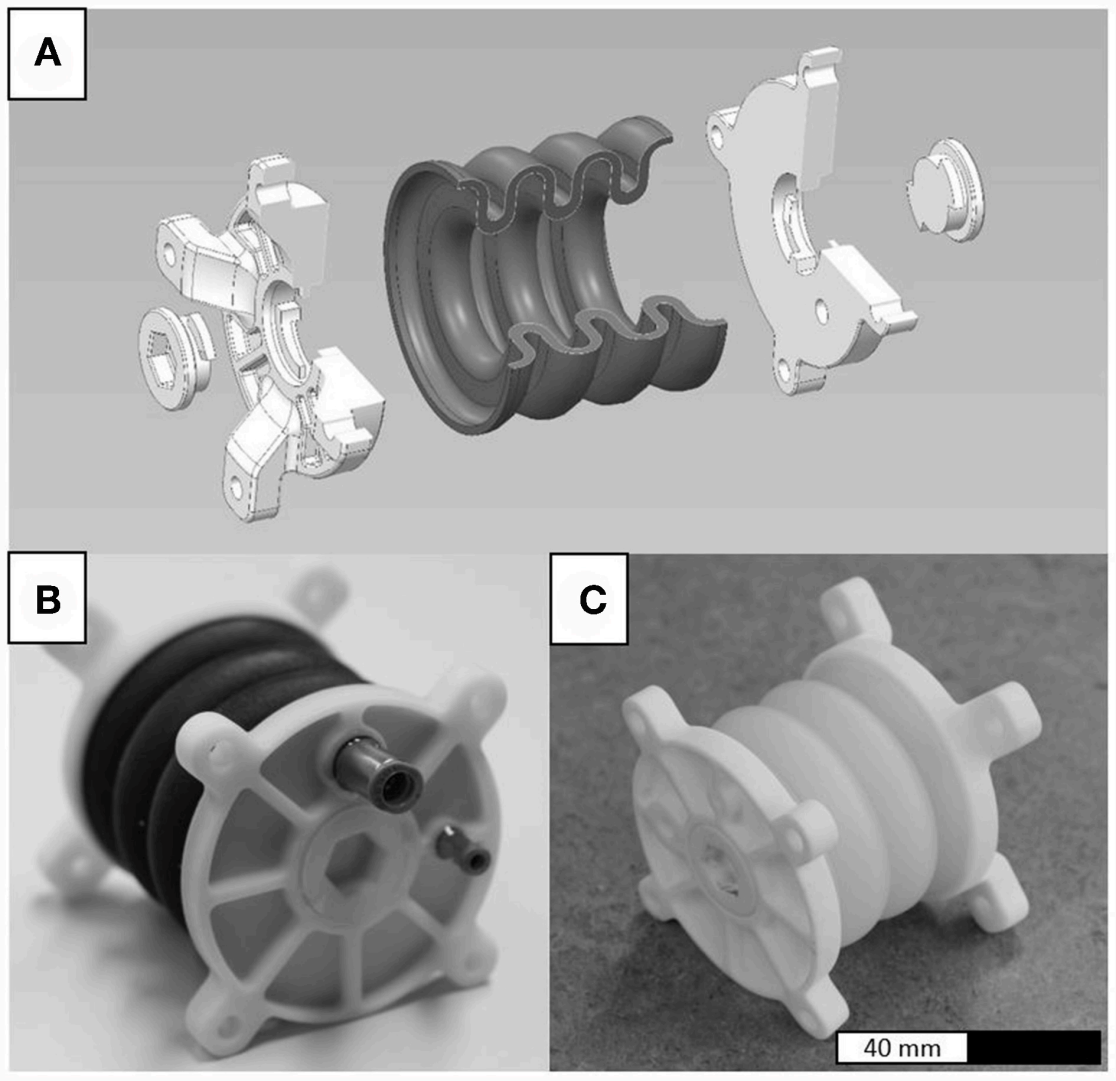
Linear bellows actuators **(A)** obtained by simultaneously printing hard and soft materials. The soft materials TangoBlackPlus™ **(B)** and Agilus30™ **(C)** were used. The total length of the actuators is 78 mm and the outer diameter of the bellows structure is 64.2 mm.

The performance requirements (e.g., force, deflection) and available space for a bellows actuator may vary significantly depending on the intended application and specific design of surrounding components. The bellows actuators described in the following are not intended for the use in a real-life application, but for the understanding of fundamental principles that may be transferred to arbitrary applications. Therefore, PolyJet printable linear bellows actuators (Figure 1) were designed that comprise a soft, elastomeric bellows structure and hard, thermosetting flanges. Holes were placed in the flanges to be able to remove the waxlike material that supports overhangs during the printing process (Figure 1A).

The bellows structure and flanges are manufactured in one multi-material piece and complemented with closing caps and pneumatic connectors. In this paper, the standard soft material TangoBlackPlus™ (TB+) (Figure 1B) and novel soft material Agilus30™ (A30) (Figure 1C) are compared. For the flanges, the standard hard material VeroWhitePlus™ (VW+) was used. Actuators containing TB+ (Figure 1B) were printed by cirp GmbH (Römerstraße 8, 71296 Heimsheim, Germany), actuators containing A30 (Figure 1C) by Stratasys^®^ Ltd. (Haim Holtsman St. 1, 7612401 Rehovot, Israel).

## Finite Elements Analysis

### Preliminary Considerations

In general, linear actuators are used to transform different sources of energy (e.g., electric, pneumatic) into mechanical energy i.e., create force and translational motion. The usable force (referred to as “effective force”) *F*_eff_ and linear deflection *x* of a pneumatic bellows actuator are dependent on the bellows geometry and material as well as the pneumatic and mechanical components of the complete actuation system. Assuming quasi-static conditions and frictionless guiding, *F*_eff_ can be expressed as a function (1) of the pressure force *F*_p_ and the structural force *F*_s_. Thereby *F*_s_ is caused by the bellows deformation.

(1) $$\begin{array}{r} {F_{\text{eff}}\;=\;F}_{\text{p}}\;-\;F_{\text{s}} \end{array}$$

*F*_p_ can easily be determined (*F*_p_ = (Δ*p*)· *A*_eff_) by multiplying the relative pressure Δ*p* (Δ*p* = *p*_i_ − *p*_a_) and the effective area *A*_eff_ of the actuators flange. The structural force *F*_s_ however, is a function of *x* and Δ*p* (and ẋ, *t* if visco-elasticity is considered) and can hardly be computed analytically. Therefore, the following sections describe a geometrical representation and a material model for elastomeric bellows structures for the implementation in finite elements analysis (FEA).

### Geometry Representation

**Figure 2:**
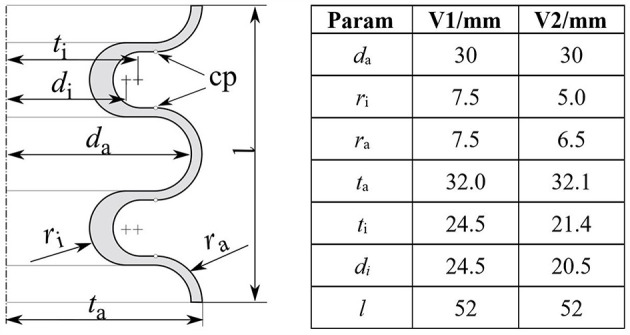
Left: parameterization of a u-shaped bellows structure with non-constant wall thickness. Displacements are read out at the control points (cp) to evaluate the distances to adjacent half-waves. Right: parameter values in mm for an initial (V1) and optimized version (V2). Thereby *d*_a_ and *l* are kept constant for comparative analysis of V1 and V2.

The elastic modulus of the flange material is in the range >1,000 MPa (Sheikhnejad et al., 2016), compared to 0,5 MPa for the elastic modulus of the bellows structure (Reiter and Major, 2011). Therefore, deformation of the flange is neglected in the following FEA i.e., the flange is solely represented by boundary conditions at the relevant nodes of the bellows structures mesh. For the bellows structure, an axisymmetric u-shaped design—comprised of parallel lines and semicircles—is chosen. The entire bellows structure with non-constant wall thickness can therefore be unambiguously defined by a planar sketch comprised of 7 design parameters (Figure 2, left). Parameter values (in mm) for an initial and optimized version are given in Figure 2 (right) and will be referred to in the subsequent paragraphs.

### Material Model

The structural force *F*_s_, exerted by the deformed bellows structure, originates in the strive of the molecular chains to return to their initial configuration. On a macroscopic level, entropic elasticity in elastomers is typically described by a strain energy function *U* (Ogden, 1997). In the polynomial form (Equation 2) (Dassault Systemes Simulia Corp., 2014), *U* is expressed as a function of the first and second invariant (*I*_1_, *I*_2_) of the left Cauchy-Green deformation tensor and—in case of compressibility—of the elastic volume strain *J*_el_ as

(2) $$U=\;\sum_{i+j=1}^{N}C_{ij}{\left(I_{1}-3\right)}^{i}{\left(I_{2}-3\right)}^{j}+\sum_{i=1}^{N}\frac{1}{D_{i}}{\left(J_{\text{el}}-1\right)}^{2i}.$$

Thereby, the material constants *C*_*ij*_ and *D*_*i*_ are related to the deviatoric and volumetric material behavior, respectively. For the FE simulations described below, the general polynomial form (Equation 2) is reduced to the first order (*N* = 1) Mooney-Rivlin form for compressible materials (Dassault Systemes Simulia Corp., 2014) in the form of

(3) $$\begin{array}{r} \text{U}=C_{10}\left(I_{1}-3\right)+C_{01}\left(I_{2}-3\right)+{\frac{1}{D_{1}}\left(J_{\text{el}}-1\right)}^{2}. \end{array}$$

The material constants that are used in the following (*C*_10_ = 0.11 MPa, *C*_01_ = 4.52 MPa, *D*_1_ = 2.28 MPa) were determined by fitting the model (Equation 3) to uniaxial tensile and compression test data of TB+ using Abaqus' (Dassault Systèmes) fitting procedure.

## Experimental Verification

**Figure 3:**
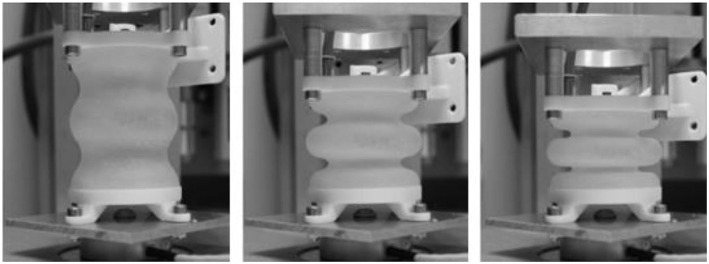
Measuring the effective force *F*_eff_ of a linear bellows actuator. Three displacement states were tested i.e., extension (left), initial (middle) and compression (right) state. Excessive extensions lead to large strains and structural failure, compression is limited due to self-contact of adjacent half-waves.

In order to validate the FEA of the bellows geometry, an actuator test bench was set up that allows to measure the effective force *F*_eff_ for given pressures Δ*p* and deflections *x*. Figure 3 shows a bellows actuator mounted to the actuator test bench at three displacement states. The applied pressure difference Δ*p* is controlled by a Festo VPPM pressure control valve (0–2 bar), *F*_eff_ is measured using a Burster 8523-50 force sensor (+/- 0-50 N) and the displacement *x* is controlled by a Festo EGSA-50-100 linear axis.

**Figure 4:**
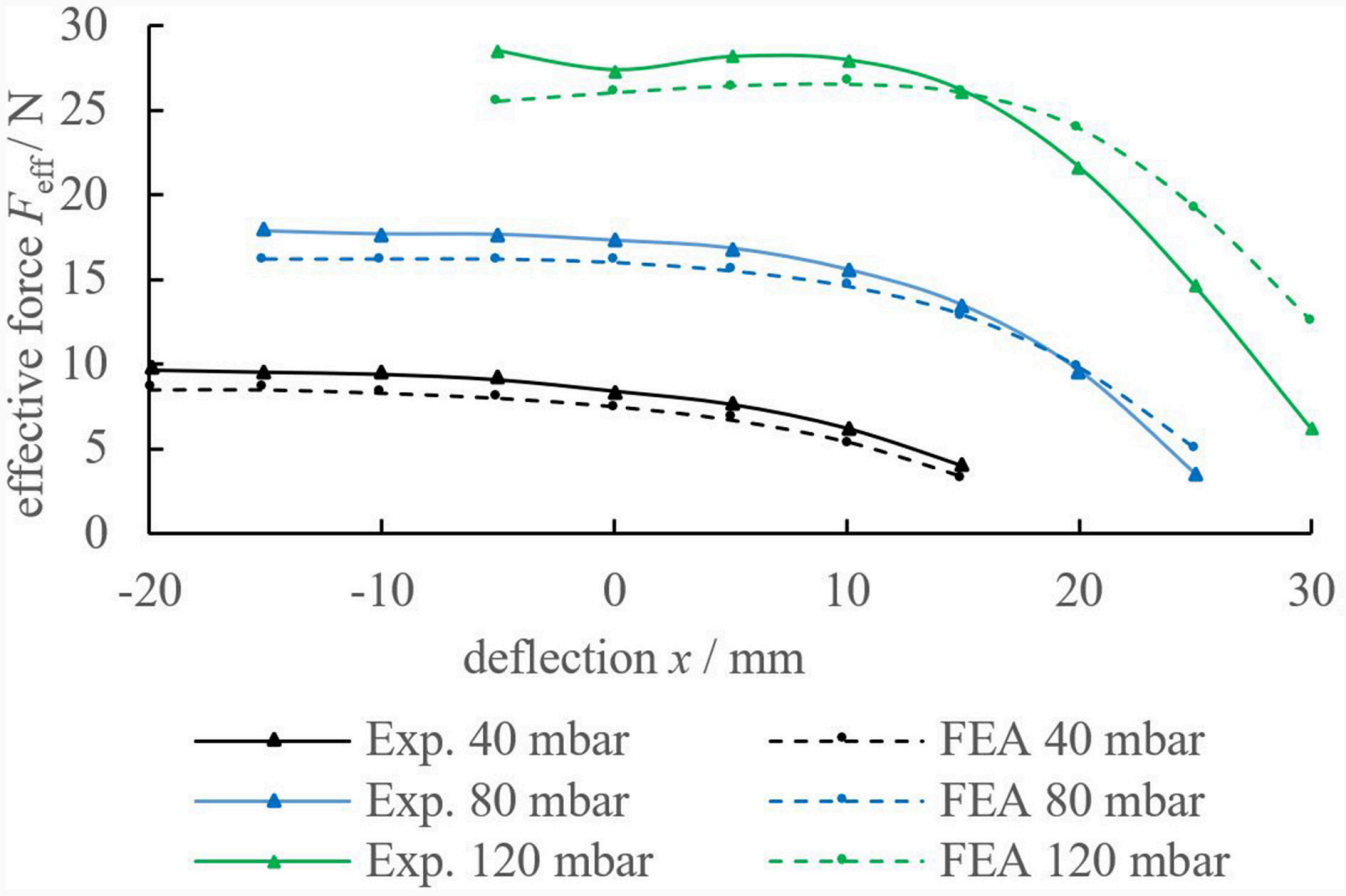
Comparison of the experimental (solid lines) and simulated (dotted line) results of the pressure and displacement dependent effective force *F*_eff_. Simulations are generally in good agreement with the experimental results. Note, that lines between measuring points are interpolated.

In the procedure, displacements were varied between −20 and 30 mm and relative pressures Δ*p* were varied from 0 to 140 mbar in 20 mbar steps. In Figure 4 the experimental (“Exp.”) and simulated (“FEA”) force-pressure-deflection characteristics are compared. The effective force was observed to increase for almost 30 s after the final displacements were reached. Therefore, measurements were taken after 30 s (relaxation is investigated closely in paragraph Investigating Relaxation in PolyJet Bellows Actuators). As expected, the effective force *F*_eff_ exerted by the actuator increases with an increase in relative pressure Δ*p* but decreases (in general) with an increase in deflection *x*. For negative deflections (*x* < 0), the effective force *F*_eff_ remains almost constant.

Noticeably, none of the lines covers the full range from −20 up to 30 mm deflection. Extension is limited at lower pressures due to a shift in the static force equilibrium (Equation 1). Compression is limited at higher pressures because the waves of the bellows geometry tend to touch adjacent waves (“self-contact”). Whereas, results from FEA are generally in good accordance with the experimental results, Figure 4 indicates, that deviations increase as a result of high pressures or elongations. This is most possibly due to the fitting range of the constitutive model. For improved accordance of simulation and experiment, material tests covering multi-axial states of stress and time-dependency are advised.

## Optimization of Bellows Geometry

### Bellows Optimization Problem

In the following paragraph an exemplary problem is solved but the shown methods are easily modifiable to other problems. In the experiments described above, repeated loadings lead to cracks in the bellows structure which can be explained by fatigue. In repeatedly strained elastomers (Gent et al., 1964; Lake and Lindley, 1964) material imperfections—also typical for AM materials (Moore et al., 2015)—cause local strain peaks that lead to the formation and propagation of microscopic cracks and eventually result in fatigue failure. To find an improved bellows design (V2), that reaches similar effective force *F*_eff_ and deflection *x* as the initial geometry (V1) but sustains an increased number of load cycles, a numerical optimization routine was developed. Thereby, maximum (logarithmic) principal strain ε_ln, max_ was considered as a fatigue life indicator (Zhou, 2016) in the strain objective function

(4) $$\begin{array}{r} Q_{\varepsilon }\left(x\right)\;=\;{\left(\varepsilon_{\ln,\;\max}\left(x\right)-\varepsilon_{\max}\right)}^{2}. \end{array}$$

Designs *x* that lead to simulated strains ε_ln, max_ larger than a reference strain ε_max_ are penalized with large objective function values. To achieve a required deflection while avoiding self-contact, the penalty functions *Q*_ld_(*x*) and *Q*_sc_(*x*) were stated analogous to *Q*_ε_(*x*) and added up to a multi-criteria objective function

(5) $$\begin{array}{r} {Q\left(x\right)\;=\;w}_{\varepsilon }\cdot Q_{\varepsilon }\left(x\right)\;+\;w_{\text{ld}}\cdot Q_{\text{ld}}\left(x\right)\;+\;w_{\text{sc}}\cdot Q_{\text{sc}}\left(x\right) \end{array}$$

with *w*_ε_, *w*_ld_, and *w*_sc_ being associated weighting factors for the compensation of units. The required effective force *F*_eff_ is implemented as a hard constraint which requires Δ*p* to be variable. The bellows structures length and effective area of the flanges were kept constant to be able to compare the effect of the optimization. A bellows design is therefore fully described by the design vector

(6) $$x={\left[r_{\text{i}}r_{\text{a}}t_{\text{i}}t_{\text{a}}d\Delta p\right]}^{\text{T}}$$

In addition, an (even) integer parameter *n*_hw_ is defined to quantify the number of half-waves that describe the bellows structure. Bounds and secondary constraints are defined (their exhaustive description is beyond the scope of this paper) in a constraint vector *g*(*n*_*hw*_, *x*) to exclude infeasible geometries. Therefore, and following (Mahl, 2015) the constraint mixed integer bellows optimization problem is

(7) $$\underset{n_{\text{hw}}}{\min}\left\{\underset{x}{\min}\left\{Q(n_{\text{hw}},\;x)|g(n_{\text{hw}},\;x)\;\;\leq \;\;0\right\}\right\}.$$

### Optimization Results and Verification

**Figure 5:**
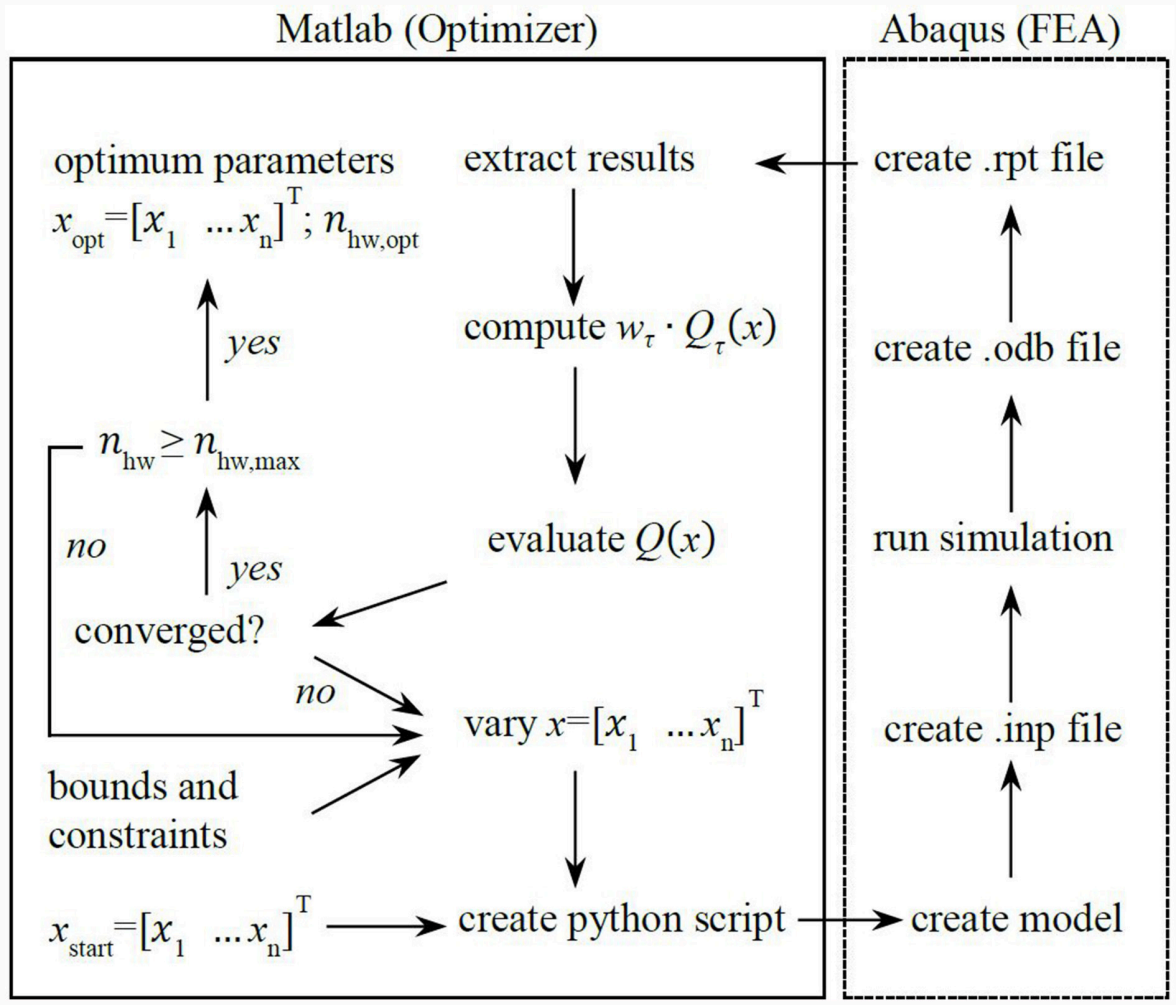
Optimization routine for the optimization of linear bellows actuators. The routine finds optimum geometry parameters and relative pressure to generate a required effective force at required displacement while minimizing the maximum principal strain in the bellows structure.

An optimization routine was realized to solve the above stated optimization problem based on the structural simulation and parameterization described above. Matlab (MathWorks) and Abaqus (Dassault Systèmes) were connected and software communication was realized by python scripts (Figure 5). The routine starts with the initial parameters *x*_start_ and *n*_hw, start_ and terminates with the optimum parameters *x*_opt_ and *n*_hw, opt_. The gradient based Matlab optimization function *fmincon* (default settings) was chosen for convenient implementation of bounds and secondary constraints. The optimization routine was run considering a maximum strain reference of ε_max_ = 0.2 and a force requirement of 12 N at 30 mm linear deflection.

**Figure 6:**
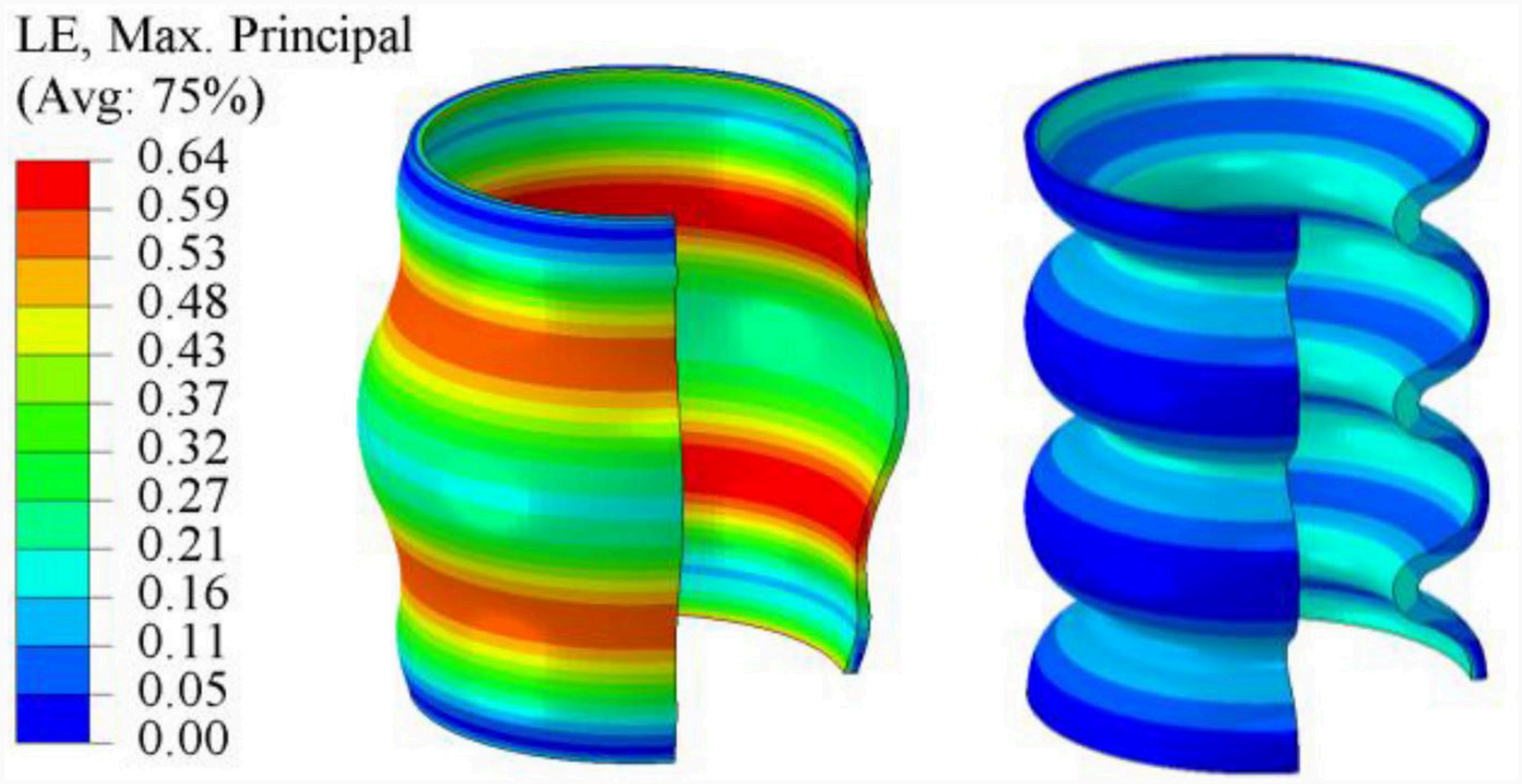
Simulated max. principal strain distribution of the initial geometry V1 (left) and optimized geometry V2 (right).

Figure 6 illustrates the simulated max. principal strain distribution of the initial (V1) and the optimized bellows geometry (V2). The corresponding shape parameters are given in Figure 2 (right). The initial geometry (V1) is described by four half-waves with constant wall thickness. To reach an effective force of 12 N at 30 mm deflection, 140 mbar are required that induce a (simulated) maximum principal strain of 65%. The optimized geometry (V2) consists of six instead of four half-waves and a non-constant wall thickness. At a linear deflection of 30 mm and an applied pressure of 100 mbar, the effective force is above 12 N and the simulated max. principal strain 24%.

**Figure 7:**
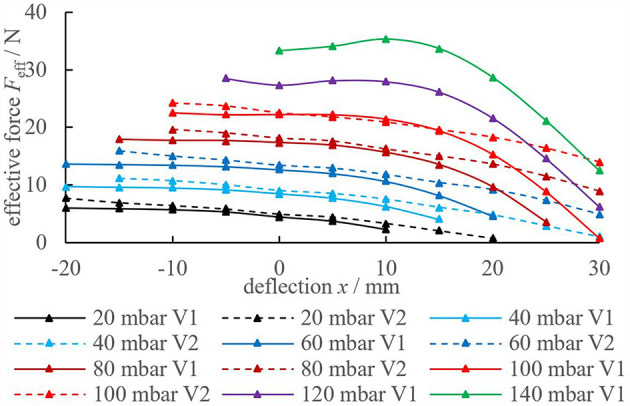
Comparison of the experimental pressure-force-deflection characteristics of the initial (V1, solid lines) vs. the optimized (V2, dotted lines) geometry. The optimized geometry (V2) requires less pressure to produce the required forces at large deflections which is a result of max. principal strain minimization. Lines between measuring points are interpolated.

In Figure 7 the pressure-force-deflection characteristics of the initial (V1) and optimized (V2) geometry are compared. For the optimized geometry (dotted lines), the effective force is significantly less dependent on the deflection, i.e., lower pressures are sufficient to generate comparatively high forces at large displacements. Considering Figure 6 in conjunction with Equation (1), it can be concluded, that the loss of effective force for increasing deflections is caused by the increasing strain in the material.

The optimized geometry (V2) satisfies the force-displacement requirements of 12 N at 30 mm. Moreover, the significant reduction of the simulated maximum principal strain (24% instead of 64%) in conjunction with fatigue data from literature (Moore et al., 2015) gives rise to assume an increased fatigue life of the new bellows geometry (V2) compared to the initial geometry (V1).

## Endurance Run

Endurance runs were performed to validate the hypothesis predicting increased fatigue life of the optimized geometry and for comparison of TB+ with a novel material. Agilus30™ (A30) is a recently released PolyJet elastomer with similar hardness range (Shore A 30–35 compared to 26–28 for TB+) as well as an increased elongation at break and tear resistance (Stratasys, Ltd.). Due to superior properties in the data-sheet (Stratasys, Ltd.), an increased fatigue life, compared to TB+, was expected. It is pointed out that the same geometries and material parameters were used for both the TB+ and A30 bellows because no sufficient material data of A30 was available at this time. Therefore, A30 results should be interpreted with care and are presented for comparative purposes.

**Figure 8:**
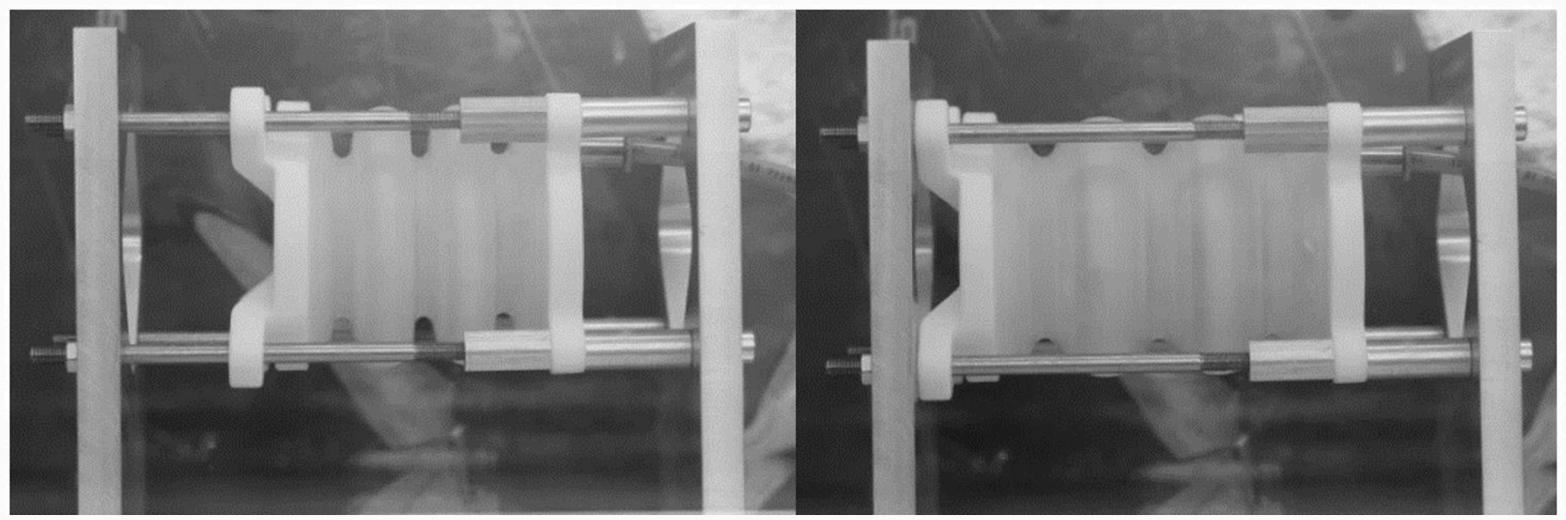
Endurance runs with bellows actuators. Initial state (left) and extension state (right). Extension is mechanically limited to 30 mm. The experiment was stopped in case a threshold volume flow was reached.

Figure 8 illustrates a bellows actuator mounted to the endurance test bench. During the test, the left side of the bellows actuator can move freely in horizontal direction and is guided by four PTFE-lubricated guiding bolts. In the endurance runs, a pressure of 140 mbar for V1 and 100 mbar for V2 was applied for 30 s. The resulting deflection was mechanically restrained to 30 mm.

Pressure was then released for another 30 s before the procedure was repeated. Volume flow was measured during the 30 s period to detect possible damage of the bellows and the experiment was stopped in case a threshold value of 2 Nl/min was exceeded.

**Figure 9:**
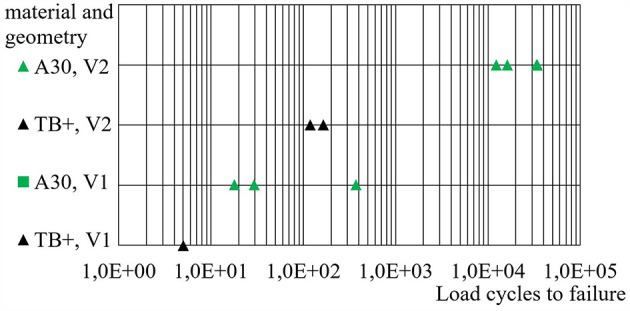
Load cycles to failure of different bellows actuators. Best results are obtained from the combination of Agilus30™ (A30) and geometry variant 2 (V2). Each data corresponds to a single endurance run.

Figure 9 contains the load cycles to failure that were reached by the bellows actuators. Cycles to failure range from below 20 (TB+, V1) to more than 30,000 (A30, V2). In average, A30 bellows with the initial geometry (V1) endured 143 load cycles and with the optimized geometry (V2) 24,104 load cycles. Even without statistical significance, the results strongly indicate that the optimized geometry (V2) sustains significantly more load cycles until failure compared to the initial geometry (V1). Moreover, we can assume that bellows made from A30 endure significantly more load cycles to failure compared to ones manufactured from TB+.

**Figure 10:**
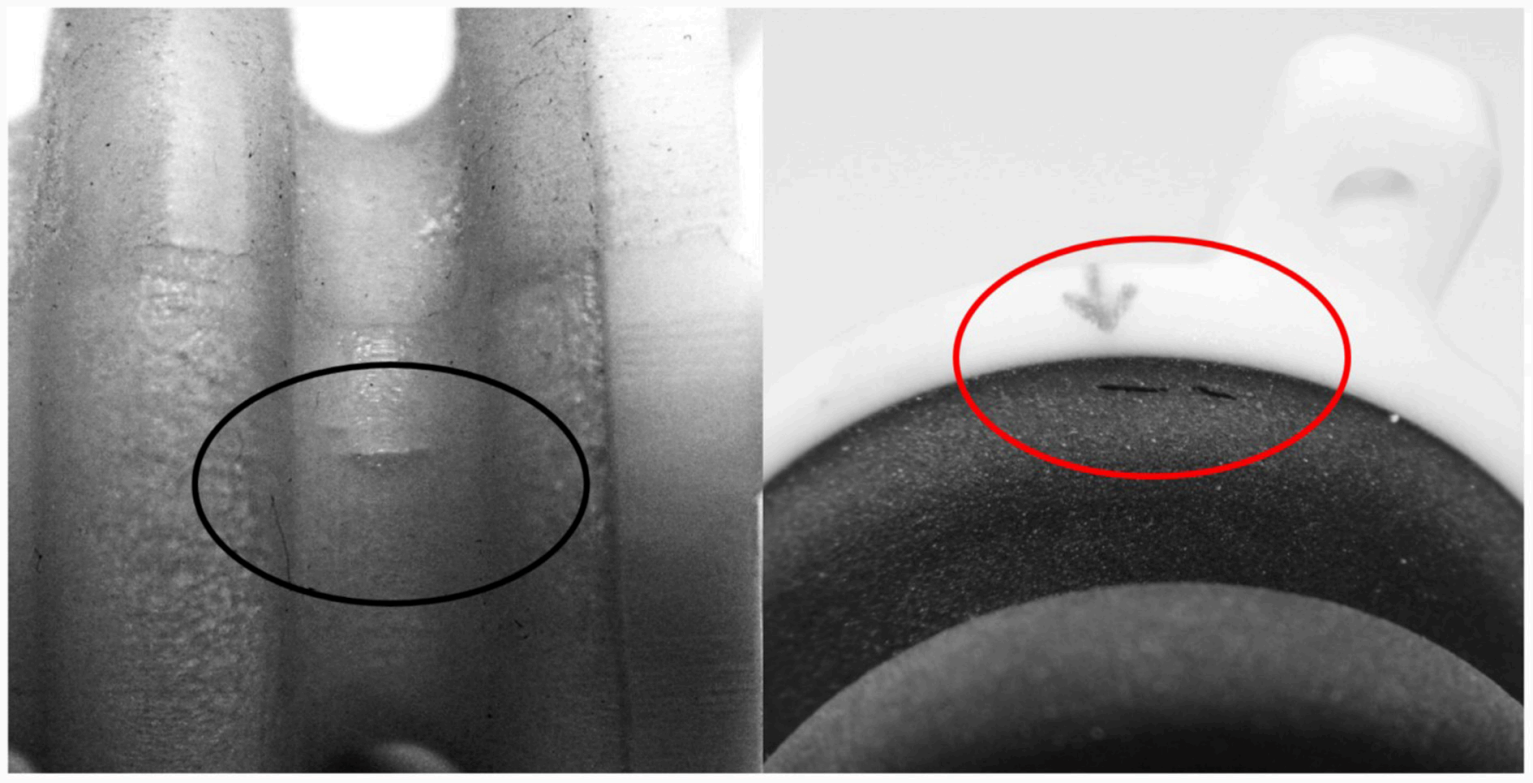
Cracks in the bellows structure are oriented in axial direction at the inner diameter of the initial geometry “V1” (left) and tangential direction next to the flange of the optimized geometry “V2” (right) which is in accordance with the locations and perpendicular to the directions of max. principal strain in the corresponding FEA.

Noticeably, different geometries lead to different but consistent modes and locations of failure. Thereby, two categories can be made for failures as shown in Figure 10. All specimens of the initial geometry (V1) failed at the inner wave due to cracks in axial direction (Figure 10, left). In the corresponding FEA max. principal strains are oriented in tangential direction at the inner diameter i.e., perpendicular to the cracks. Specimens of the optimized geometry (V2) consistently failed in tangential direction (i.e., perpendicular to axial strains) near to the flanges as illustrated in Figure 10 (right). Location and direction of failure are consistent with the simulated max. principal strain distribution obtained from FEA.

There are multiple possibilities to further increase the fatigue life of the bellows actuators described above. The specific geometric representation as described in Figure 2 presents a compromise between the dimensions (and size) of the solution space and cost (time for development of the routine itself and computation time). Further optimization could be realized by choosing a more complex parameterization. Note, that the rotationally symmetric design was chosen here in order to minimize the dimensions of the optimization problem. If this simplification is omitted, the effective flange area could be increased without an increase in the main dimensions of the actuator.

## Investigating Relaxation in PolyJet Bellows Actuators

### Motivation for Investigating the Relaxation in PolyJet Bellows Actuators

The accuracy of a pneumatic robotic system depends in many cases on the accuracy with which its actuators are modeled. This is, because in state-of-the-art model-based controllers a physical model of the system is utilized to compute the desired pressures. In bellows actuators, the pressurization or forced motion of the actuator implies a deformation of the bellows structure i.e., different pressure or motion profiles result in different strains/strain rates in the bellows material. Depending on the specific application or task of a light-weight robot, cycle times may range from fractions of a second to several seconds or minutes. During the experiments described in the previous paragraphs we experienced that the PolyJet printed bellows react significantly time dependent to applied pressures and forced deformations. In paragraph Experimental Verification, this fact was accounted for by waiting until the force responses of the structures had stabilized (i.e., 30 s in Figures 4, 7) before measurements were taken. However, this procedure is impractical for an actual robotic application and implications for structural simulation have to be evaluated. Thereby, time dependent mechanical behavior is typical for elastomeric structures (Saccomandi and Ogden, 2004; Bergström, 2015) and originates mainly from the rearrangement of the molecular chains (entropic elasticity) induced by deformation. Several publications confirm our qualitative experiences concerning the time dependency of PolyJet materials in general (Blanco et al., 2014; Zhang and Albert, 2016) and—most important—the PolyJet elastomers TangoBlack™ (a predecessor of TB+) (Kundera and Bochnia, 2014), and A30 (Akbari et al., 2018). However, to our knowledge no closer investigations on the time dependent behavior of A30 bellows structures were published yet. Therefore, in the following paragraphs the relaxation behavior of A30 bellows structures is investigated. Results are intended to make an initial assessment of the significance of time dependency for (soft) robotic applications and will be used for the validation of visco-elastic material models in the future.

### Redesign of Bellows Actuators for Relaxation Testing

**Figure 11:**
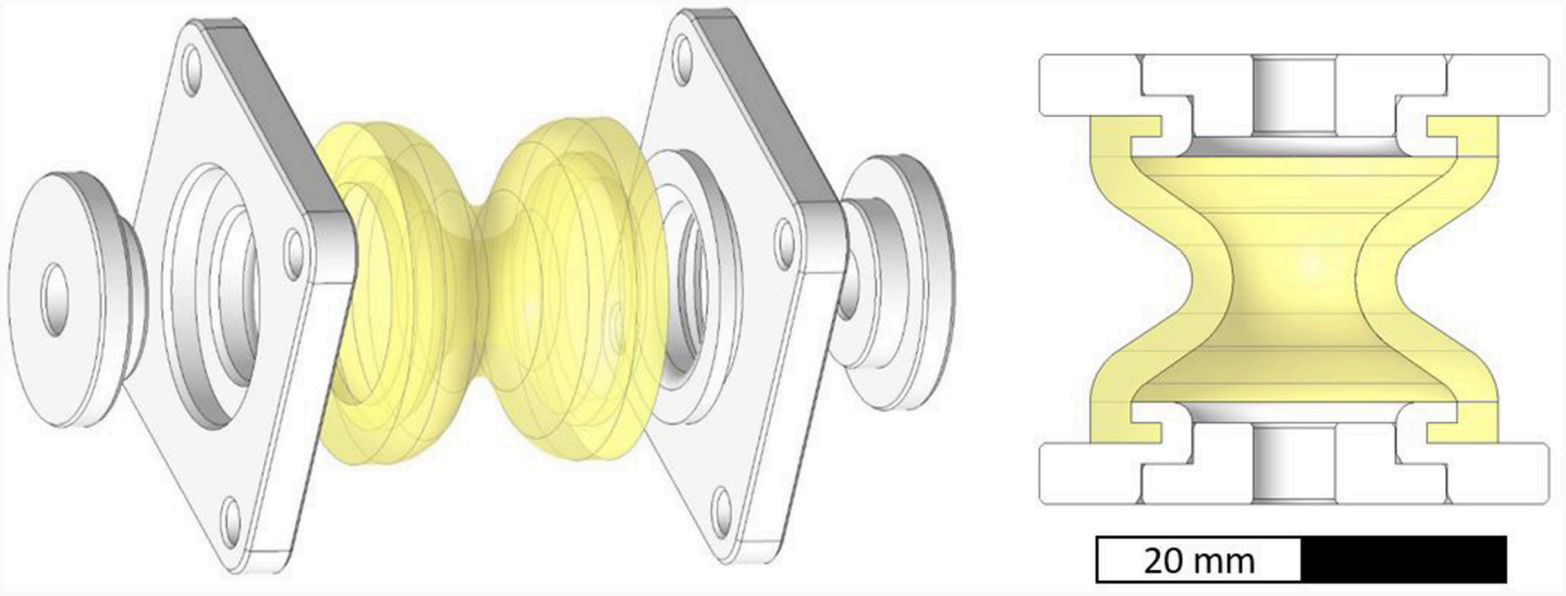
Exploded (left) and sectional (right) view of PolyJet printed bellows structure for relaxation testing. The bellows (yellow) and square shaped flanges (white) are printed into one multi-material part.

In order to increase cost-efficiency, new test geometries (Figure 11) were designed that are considerably smaller (total length of 22 mm instead of 78 mm) than the ones described in Design. Regarding future research, a simple bellows geometry with two halve waves was chosen that can also be molded using a two-part core (e.g., for silicone molding).

The soft bellows structure (yellow) is printed together with two rigid square shaped flanges (white). Circular end caps (white) are printed separately and mounted after the support material is removed from the bellows chamber. The bellows actuators were printed by cirp GmbH (Römerstraße 8, 71296 Heimsheim, Germany). All parts investigated in paragraph Investigating Relaxation in PolyJet Bellows Actuators were printed with matte finish and oriented with the main axis parallel to the printing platform.

### Experimental Setup and Relaxation Testing Procedure

Two load cases were applied, in order to investigate the relaxation behavior of the bellows actuators (Figure 11).

Load case “Deflection”: starting from the initial length of the actuator (22 mm), the upper flange was pulled with a rate of 8 mm/s until a linear deflection of 4 mm was reached. The deflection was maintained for 90 s and the (retracting) force exerted by the deflected actuator was monitored. No pressure differential was applied in load case “Deflection”.

**Figure 12:**
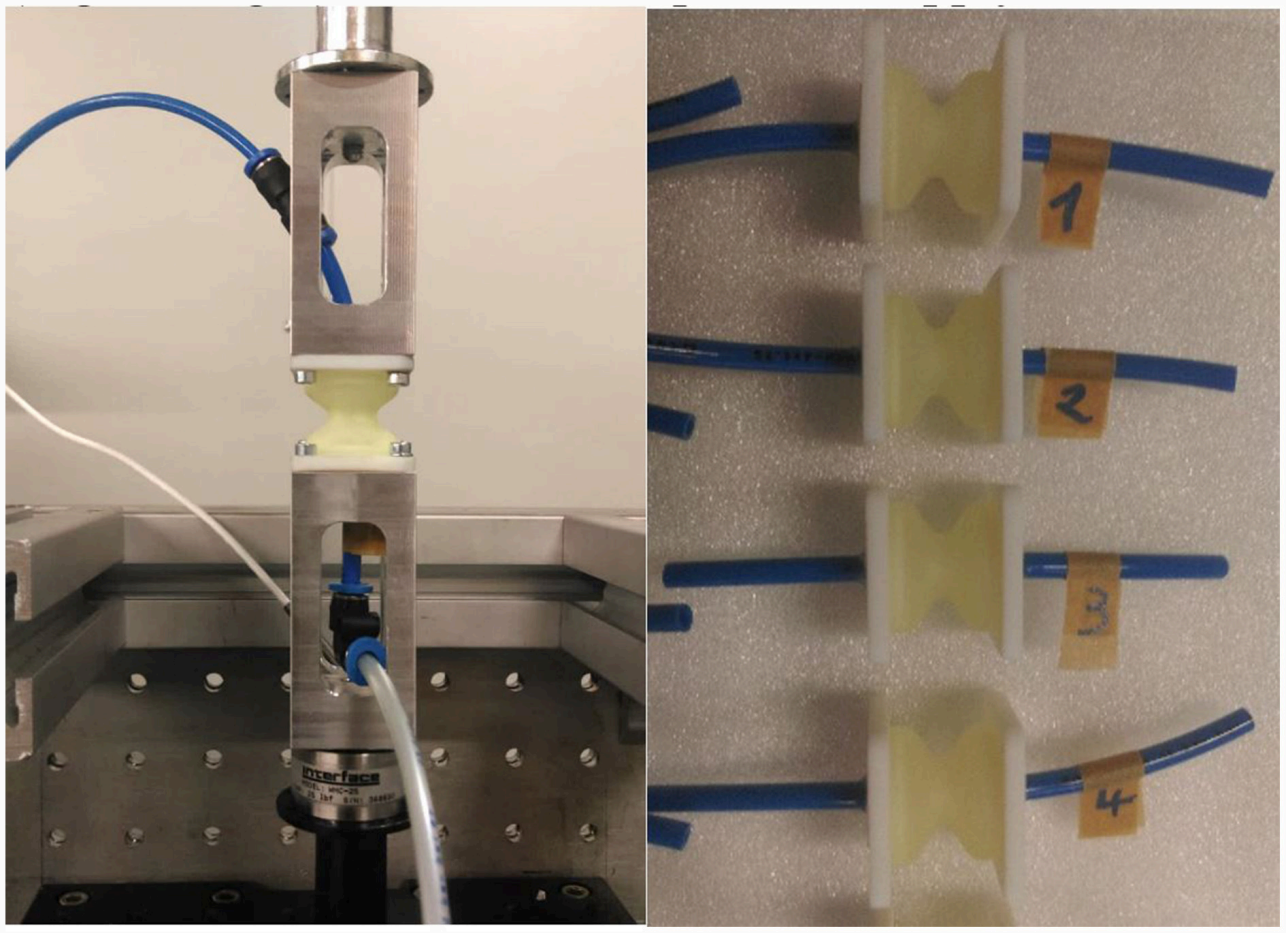
Relaxation tests with A30 bellows actuators. Left: pneumatic actuator mounted to the test bench, air hoses for pressure supply/sensor and load cell. Right: bellows actuators tested for this publication.

Load case “Pressurization”: starting from ambient pressure in the bellows actuator, a pressure step of +0.4 bar was applied by quickly opening a shut-off valve. Deflection was completely suppressed and the increased relative pressure was maintained for at least 90 s. The exerted force of the pressurized actuator was monitored. In order to apply the described loads and monitor the applied and resulting parameters, a test setup (Figure 12, left) was build up. Pneumatic hoses were attached to the actuators (Figure 12, right) to connect the pressure supply and sensor.

The test bench (Bose Corp., ElectroForce Systems Group, MN, US) comprises a linear actuator for monotonic tests, a WMC-25 load cell (Interface Inc. AZ, US) and a controller unit and workstation. WinTest® DMA software (Bose Corp.) was utilized to perform and analyze the experiments. For presetting and applying the preselected pressure, a Festo LRP-1/4-4 precision pressure (Festo AG & Co. KG, GER) regulator and a manual Festo shut-off valve were used. Pressure was measured using a Keller 21 PY (Keller AG, CH) pressure transmitter.

A total of four equal specimen was tested. Thereby, each specimen was tested two consecutive times in one load case. The testing sequence was varied according to Table 1 in order to account for possible irreversible effects, such as plastic deformation or cracks.

**Table 1:** Sequence of relaxation tests.

**Nr**.	**Test 1**	**Test 2**	**Test 3**	**Test 4**
1	Pressurization	Pressurization	Deflection	Deflection
2	Pressurization	Pressurization	Deflection	Deflection
3	Deflection	Deflection	Pressurization	Pressurization
4	Deflection	Deflection	Pressurization	Pressurization

### Results and Interpretation

**Figure 13:**
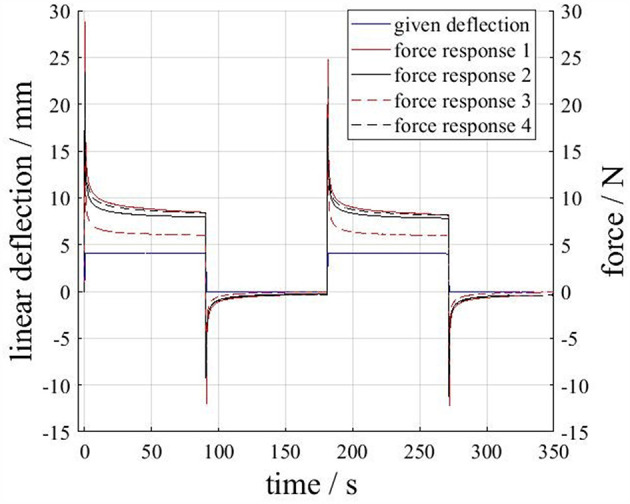
Actuator force responses due to an applied deflection. The applied deflection profile is plotted (blue) for the first test run only, but is virtually the same for the other test runs.

Figure 13 displays the given deflection and force responses of the four actuators in the load case “Deflection”. As can be seen, the rapidly applied displacement (4 mm in 0,5 s) leads to pronounced peak forces for all actuators. During times of constant displacement, the amount of the actuator forces decreases and asymptotically approaches a plateau. The retraction of the test bench to the initial length causes an opposed force. Whereas, the force responses of the actuators 1,2 and 4 are similar, the force response of actuator 3 differs significantly in amplitude.

**Figure 14:**
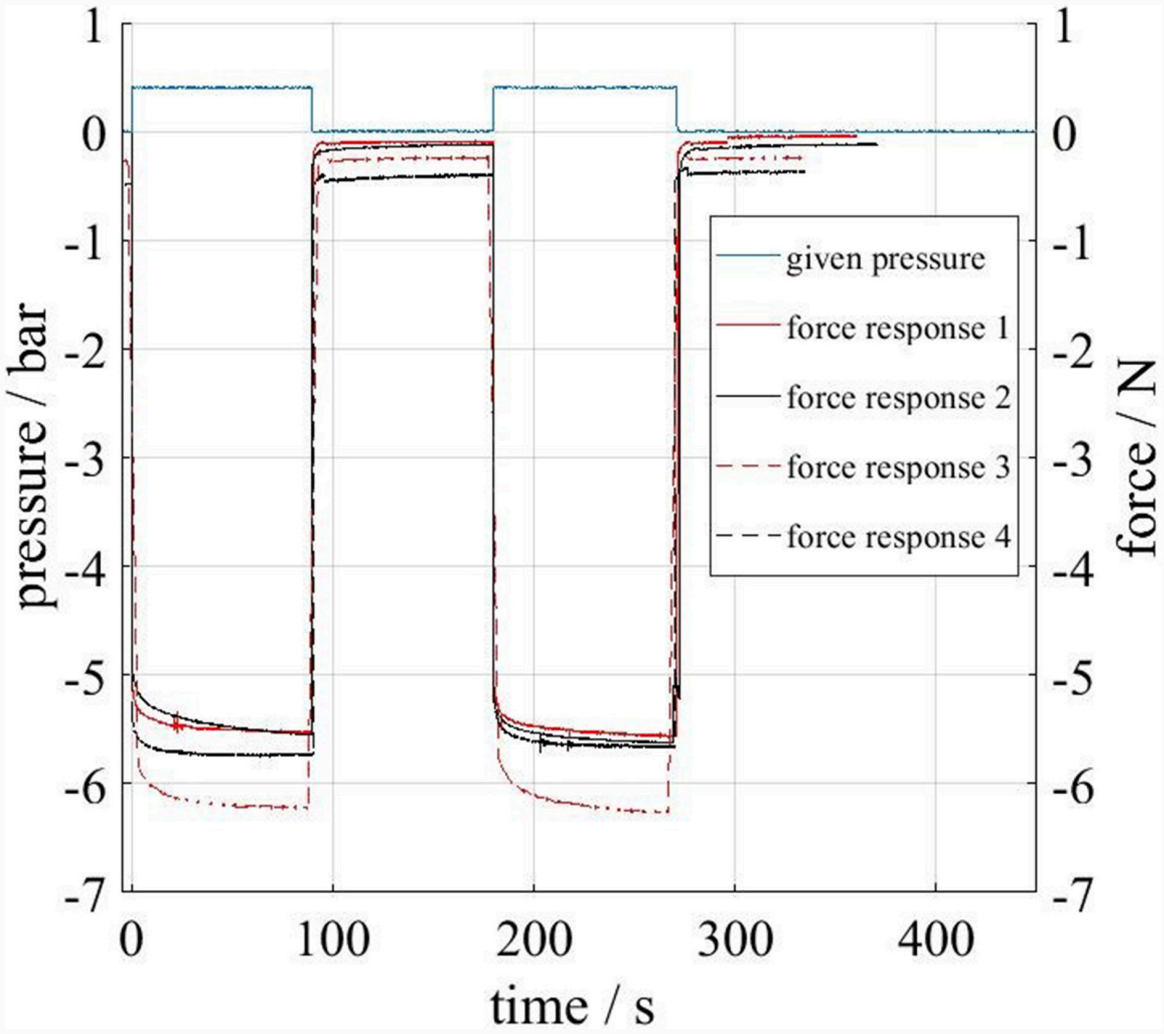
Actuator force responses due to an applied pressure. For the sake of clarity, the applied pressure profile is plotted (blue) for the test run with the first actuator only.

In Figure 14 the force responses of the four actuators in the load case “pressurization” are displayed. The rapidly applied pressure step (+0.4 bar; plotted for the test with the first actuator only) leads to a nominal increase in actuator force. During the intervals of constant pressure, the force responses asymptotically approach a plateau. The force response of actuator 3 is significantly larger (in amount) than the other responses. The reason for this deviation is not known. However, in combination with the results from Figure 13 it can be concluded, that actuator 3 poses less resistance to applied loads (i.e., has a “softer” structure) which results in a lower measured force under deflection but a larger measured force amount under pressurization. Further investigations including larger numbers of specimen have to clarify this.

Nevertheless, two interesting observations can be made from Figures 13, 14. First: all force responses are significantly time dependent, e.g., peak forces in Figure 13 reach over 20 N but decrease quickly below 10 N. Second: the deliberate pre-stretching during the loadcases applied in our tests seems to have little effect on the structures stiffnesses in subsequent tests. This can be concluded from the fact that consecutive tests lead to similar force responses (Figures 13, 14) and that no clear distinction can be made between pre-stretched and non-pre-stretched actuators in Figure 13. The observed time dependency is typical for elastomers and in line with our qualitative expectations. However, the described results may be used to build mathematical models of the actuators time dependent behavior and serve as a starting point for standardized cyclic and rate dependent tests for the calibration of visco-elastic material models.

For the FEA and optimization of the bellows actuators in paragraphs Finite Elements Analysis–Optimization of Bellows Geometry, the constitutive behavior was modeled as time independent which represents a major simplification considering the results presented in this paragraph. In this context it is also important to note, that all presented data regarding strain is simulative and we have no evidence of the actual level of strains in the structure. However, the results of the endurance runs in paragraph Endurance Run proof, that the shape optimization lead to an significantly increased fatigue life. Moreover, the comparison of simulative strain maxima (Figure 6) and experimental failure modes and locations (Figure 10) indicates a good significance of the FEA considering the qualitative strain distribution in the structure.

## Conclusion

Additively manufactured bellows actuators pose an interesting option for the actuation of future robotic systems as their structural behavior is highly tunable by shape and material. This paper contains the design, shape optimization and experimental investigations of pneumatic PolyJet bellows actuators. First, multi-material bellows actuators were designed using VeroWhitePlus™ material for the rigid flanges and TangoBlackPlus™ (TB+)/Agilus30™ (A30) for the soft bellows structure. Then, the bellows structural behavior was analyzed by finite elements analysis. A numerical optimization strategy was developed and the effect of geometry optimization and material selection on the sustainable number of load cycles was investigated. Results strongly indicate that the proposed design strategy—based on a multi-criteria optimization routine—leads to significantly improved fatigue life. Moreover, A30 bellows withstood significantly higher numbers of repeated loadings than TB+ bellows. Modes and locations of failure largely correspond with finite elements analysis. In this extended version, the better performing material (A30) was investigated more closely. Therefore, relaxation test were carried out using redesigned test structures. The findings confirm the significance of time dependency of A30 material behavior. Results contribute to the understanding of the mechanical behavior of PolyJet elastomers under static, dynamic and repeated loadings, such as present in printed robots.

## Author Contributions

GD coordinated the activities at Festo regarding mechanics in the DIMAP project. This included literature research, coordinating the tests, interpreting the results as well as planning and writing the conference paper and extended version. SG realized the shape optimization routine and component tests for the conference paper. AH supported the DIMAP project at Festo and coordinated the activities regarding feedback control for the paper. ZM initiated the DIMAP project at IPPE and coordinated all material and component tests at IPPE.

### Conflict of Interest Statement

During the period in which the described work was done, GD, SG, and AH were employed by Festo AG. & Co. KG., Esslingen, Germany and ZM was employed by Johannes Kepler University, Linz, Austria. Stratasys, PolyJet, TangoBlackPlus, TangoBlack, VeroWhitePlus, and Agilus30 are trademarks of the company Stratasys Ltd. (Rehovot, Israel) in some countries.
